# Anti-inflammatory and antioxidant traditional Chinese Medicine in treatment and prevention of osteoporosis

**DOI:** 10.3389/fphar.2023.1203767

**Published:** 2023-06-27

**Authors:** Qian Li, Ciqiu Tian, Xiangjie Liu, Dinglin Li, Hao Liu

**Affiliations:** ^1^ Laboratory of Metabolic Abnormalities and Vascular Aging, Liyuan Hospital Affiliated to Huazhong University of Science and Technology, Department of Integrated Chinese and Western Medicine, City Wuhan, Hubei Province, China; ^2^ Hubei University of Chinese Medicine, City Wuhan, Hubei Province, China; ^3^ Liyuan Hospital Affiliated to Huazhong University of Science and Technology, Geriatric Department, City Wuhan, Hubei Province, China

**Keywords:** inflammatory, oxidative stress, osteoporosis, traditional Chinese medicine, network pharmacology

## Abstract

A metabolic bone disorder called osteoporosis is characterized by decreased bone mass and compromised microarchitecture. This condition can deteriorate bones and raise the risk of fractures. The two main causes of osteoporosis are an increase in osteoclast activity or quantity and a decrease in osteoblast viability. Numerous mechanisms, including estrogen shortage, aging, chemical agents, and decreased mechanical loads, have been linked to osteoporosis. Inflammation and oxidative stress have recently been linked to osteoporosis, according to an increasing number of studies. The two primary medications used to treat osteoporosis at the moment are bisphosphonates and selective estrogen receptor modulators (SERMs). These medications work well for osteoporosis brought on by aging and estrogen deprivation, however, they do not target inflammation and oxidative stress-induced osteoporosis. In addition, these drugs have some limitations that are attributed to various side effects that have not been overcome. Traditional Chinese medicine (TCM) has been applied in osteoporosis for many years and has a high safety profile. Therefore, in this review, literature related to botanical drugs that have an effect on inflammation and oxidative stress-induced osteoporosis was searched for. Moreover, the pharmacologically active ingredients of these herbs and the pathways were discussed and may contribute to the discovery of more safe and effective drugs for the treatment of osteoporosis.

## 1 Introduction

Osteoporosis is a chronic metabolic disease that is connected with estrogen deficiency, aging, chemical agents, decreased mechanical load (Nai-Dan et al., 2016), inflammatory (BoCun-Yu, 2022), oxidative stress (Jeff et al., 2021), and other factors. The prevalence of osteoporosis is widespread and it affects approximately 75 million people and an estimated 10 million women worldwide (Kerri et al., 2011). Hip fractures due to osteoporosis are expected to increase to 6.3 million by the year 2050 (Hong-Tao et al., 2006). Osteoporosis is characterized by decreased bone mass and damaged bone structure, which predisposes one to variations of fractures. Statistically, one in three older women is affected in this way (SalilaDorothy and Ethel, 2014). Clinically approved drugs for the treatment and prevention of osteoporosis primarily include bisphosphonates, selective estrogen receptor modulators (SERMs), and so on. However, gastrointestinal reaction, renal toxicity, and other side effects may be induced due to the poor bioavailability and low permeability of bisphosphonates (Suman et al., 2021). SERMs may increase the incidence rate of breast and endometrial cancer (Rossouw et al., 2002). Furthermore, given that these drugs are commonly used for estrogen deficiency, aging, and certain hormone-induced osteoporosis, studies on the specific effect on inflammation and oxidative stress-induced osteoporosis are relatively. scarce Therefore, potential drugs with fewer side effects and more specific treatments should be actively sought to make up for the shortcomings of existing drug therapies.

Traditional Chinese medicine (TCM) has been broadly used in treating multifarious diseases on account of its tiny adverse reaction. Many Chinese medicines have been shown to cure osteoporosis caused by estrogen deficiency, aging, glucocorticoid, and decreased mechanical load (Emmanuel et al., 2014; Yinbo et al., 2015; LinBeibei et al., 2019; Joon-Ho et al., 2022). Moreover, more Chinese botanical drugs have been proved to be applicable in treating osteoporosis on account of their anti-inflammatory and antioxidant effects (Xiaobin et al., 2020; Mao et al., 2021; Cheng et al., 2022). In order to create greater efficacy and reduce side effects, various botanical drugs are mixed and boiled into decoctions according to the prescription theory rather than a single TCM (Zhen G. et al., 2013). Therefore, it is difficult to figure out which component of the medicine is responsible for the treatment, which also limits the popularity of TCM worldwide. However, with the development of network pharmacology, the active ingredients of drugs and their target genes can be excavated by public website information. In additional, bioinformatics analysis can be employed to reveal the pathways through which botanical drugs may exert their anti-osteoporosis effects.

This review summarizes Chinese botanical drugs that can be utilized to treat osteoporosis through their anti-inflammatory and antioxidant properties and provides an in-depth analysis of their active ingredients. Finally, combined with network pharmacology and bioinformatics indications, the pathways that may be involved in the anti-osteoporosis effects were estimated. This may help to better explore its mechanisms and develop potential therapeutic agents.

## 2 Oxidative stress and inflammation induced osteoporosis

Oxidative stress is a pathological process in which cells produce excessive reactive oxygen species (ROS) that cannot be neutralized by tissues and antioxidant systems, causing damage to cellular molecules such as DNA, proteins, and lipids (Tarique et al., 2016). Inflammation is a defense mechanism when the body is infected by microorganisms or viruses, and exposed to allergens or any other foreign sources (Palanisamy et al., 2016). Several studies have linked inflammation and oxidative stress to the progress of cancer, mental illness, and cardiovascular disease (Simone et al., 2010; Daniel et al., 2017; Noemí et al., 2017; Bee and Mohd, 2019). Oxidative stress mediates the occurrence of diseases through two main mechanisms. One is lipid peroxidation mediated by hydroxyl radical (•OH) and peroxynitrite (ONOO−). The second is that the activity of antioxidant enzymes such as glutathione, thioredoxin, and NADPH is inhibited (Henry and Hongqiao, 2021). The pathogenesis of inflammation mainly includes interleukin, tumor necrosis factor, and other inflammatory factors that change the cell environment; the interaction between inflammatory factors and oxidation reaction damages DNA and affects the homeostasis of endoplasmic reticulum, mitochondria, and other organelles; and activation of inflammasome (Prof and Prof, 2001; Ji-Chun et al., 2016; Myles et al., 2016; Gregory et al., 2021; Paul N Cori and Russell, 2022).

**FIGURE 1:**
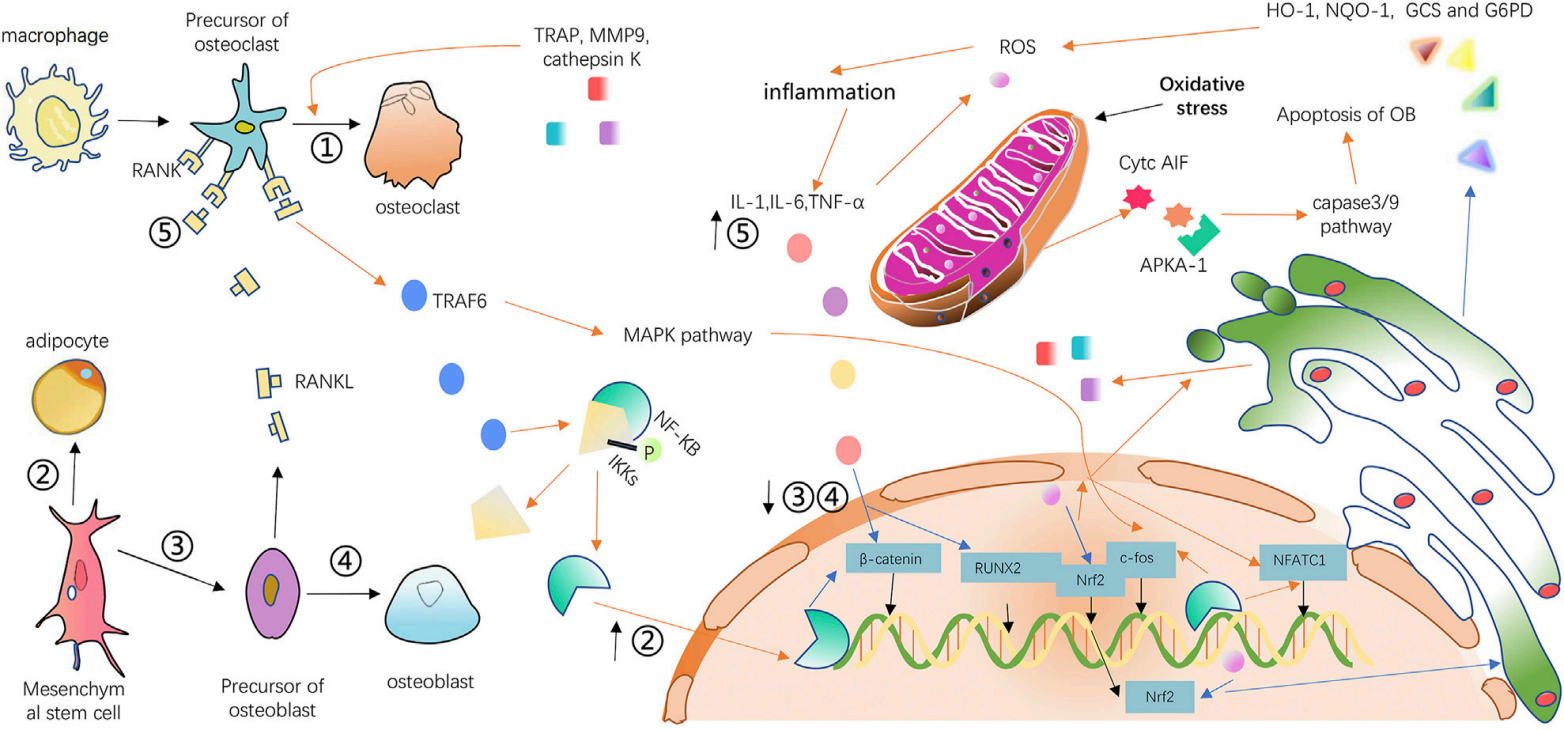
Mechanism diagram of osteoporosis mediated by inflammation and oxidative stress. The orange arrows indicate that paths have been enhanced, and the blue arrows represent that paths process has been weakened. The upward black arrow next to the number indicates that the path has been reinforced, while the downward arrow represents that the path corresponding to the number has been weakened.

Recently, increasing research has demonstrated that inflammation and oxidative stress are highly associated with osteoarticular disease, especially osteoporosis (Jeff et al., 2021; BoCun-Yu, 2022). The increase of inflammatory factors such as interleukin-1 (IL-1), interleukin-6 (IL-6), and tumor necrosis factor-α (TNF-α) during the inflammatory response can inhibit the activity and formation of osteoblasts by suppressing the expression of runt-related transcription factor 2 (RUNX2, a regulator of osteoblast differentiation) and stimulate the release of RANKL to promote the activity and formation of osteoclasts. Excessive RANKL binding with RANK will lead to the recruitment of TNF receptor-associated factor 6 (TRAF6) (Yunpeng et al., 2013; Izawa et al., 2014). As a result, the downstream pathway including nuclear factor kappa-light-chain-enhancer of activated B Cells (NF-κB), c-hJun N-terminal kinase (JNK), ERK (extracellular regulated protein kinases)and p38 will be activated and the expression of osteoclast transcription factors such as proto-oncogene c-fos and nuclear factor of activated T Cells, cytoplasmic, calcineurin-dependent 1 (NFATc1) will be further increased (Lin KM. et al., 2019; Janhavi et al., 2022). The increased expression of transcription factors induces the expression of triiodothyronine receptor auxiliary protein (TRAP), cathepsin K, and matrix metalloprotein (MMP9), which are specific to osteoclasts (Hiroyuki et al., 2016). The overexpression of the Wnt family such as Wnt10a and Wnt10b greatly restrains lipogenic differentiation and promotes osteogenic differentiation of mesenchymal stem cells (MSC) through a canonical β-catenin-dependent pathway (Cawthorn et al., 2012). However, TNF-α has the ability to relieve the osteoblastogenic effects of canonical Wnt signaling. Activation of NF-Kb is attributed to the upregulation of smurf1 and smurf2, thus promoting the degradation of β-catenin (Jia et al., 2013). In addition, inflammatory cytokines also activate the NF-κB pathway by stimulating IκB kinases (IKKs) phosphorylation and allowing NF-κB and IKKs complexes to disintegrate into the nucleus (Jeff et al., 2021). Excessive ROS produced by oxidative stress can promote bone loss and contribute to osteoporosis (Teresa et al., 2023). ROS can enhance the permeability of the mitochondrial membrane and release cytochrome C (Cytc) and apoptosis-inducing factors (AIF) inside (Rui et al., 2020; Bianca et al., 2021). These factors, when combined with apoptotic protein kinase activator-1 (APKA-1), activate the caspase-9 and caspase-3 pathways in the cytoplasm, resulting in osteoblast apoptosis (Keda et al., 2022). In order to consume excessive ROS produced by oxidative stress, cells will activate various pathways resulting in inflammation, which in turn will activate NF-κB and mitogen-activated protein kinase (MAPK) pathways which include JNK, ERK, and P38 contributing to increased osteoclast activity and generation (Kerri et al., 2011; Dinesh et al., 2017; Zhikun et al., 2018). Nuclear factor E2-related factor 2 (Nrf2) is a transcription factor for a variety of cell protective enzymes such as heme oxygenase-1 (HO-1), NADPH: quinone oxidoreductase 1 (NQO-1), γ-glutamylcysteine synthetase (GCS), and glucose 6-phosphate dehydrogenase (G6PD) (Agnieszka et al., 2016). Oxidative stress reduces the nuclear localization of Nrf2 and thus reduces the production of cell protective enzymes. As a consequence, oxidative stress is aggravated; in contrast, low expression of Nrf2 directly causes increased osteoclast generation (Teresa et al., 2023). The mechanism of osteoporosis induced by oxidative stress and inflammation is shown in Figure 1.

## 3 Single TCM with anti-inflammatory and antioxidant effects in osteoporosis

TCM mainly includes animal medicine, plant medicine, and shell medicine. The primary parts of animal drugs were the whole scale, viscera production, horn, or the whole animal body. The medicinal parts of plant medicine include the heels, stem, leaf, seed, and flower. Shell drugs are generally in the form of ground powder decoctions. Since the use of animal drugs is against the humanitarian spirit and shell drugs need to be highly processed, plant drugs have become predominant in TCM, the form of which is decocted in water In the course of thousands of years of application of TCM, TCM has been used in accordance with TCM theories such as Yin and Yang theory, five elements theory (Yan Zou, from 324 BC to 250 BC), Qi, blood and body fluid theory (“Emperor Neijing”, from Qin dynasty to Han Dynasty), eight principles of dialectics (Zhongjing Zhang, Han dynasty, “Treatise on Febrile Disease”) and so on, thus deviating from modern pharmacology knowledge to a certain extent. In recent years, with the extensive application of TCM and the continuous development of TCM pharmacology, there have been specialized websites for systematic pharmacological analysis and induction of TCM ingredients (https://old.tcmsp-e.com/tcmsp.php). Among them, many TCM have been found to have therapeutic effects on a variety of diseases due to their anti-inflammatory and antioxidant activities. Such as Alzheimer, cancer, stroke, liver diseases, and osteoporosis (Jia et al., 2016; Kathy et al., 2016; Yangyang H. et al., 2020; Hansen et al., 2020; Ting et al., 2021; Yi and Meng, 2022). Here we summarize the botanical drugs that can be utilized to treat osteoporosis (Table 1). The pharmacokinetics of the active ingredients of Chinese medicines is shown in Table 2.

**TABLE 1:** Single TCM in treating osteoporosis.

TCM	Medicine composition	Modeling and treatment modalities	Effects on bones	References
Epimedium brevicornu Maxim	Icariin (flvonoid glucoside)	Iron overload MSCs model induced by ferric ammonium citrate. (MSCs co-cultured with ferric ammonium citrate for 24 or 48 h)	Promoted the differentiation of MSCs into osteoblasts	Xudong et al. (2019)
Epimedium brevicornu Maxim	Icariin	Iron overloaded mice model induced by intraperitoneal injection of iron dextran, Icariin 200 mg/kg by gavage daily for 2 months. Iron overloaded osteoblast precursor cell model induced by iron (III) chloride (osteoblast precursor cells co-cultured with iron III) chloride for 24 h)	Alleviated bone loss	Xingzhi et al. (2019)
Epimedium brevicornu Maxim	Icariin	Mice calvarial model of titanium particle- induced osteolysis, low- and high-icariin groups were gavage-fed with icariin at 0.1 or 0.3 mg/g/day, respectively	Suppressed the formation and activation of osteoclast	Hongguo et al. (2015)
Epimedium brevicornu Maxim	Icariin	Osteoclast and osteoblast models of inflammatory and oxidative stress induced by LPS. Osteoclast or osteoblast co-cultured with Icariin for 6 days	Suppressed osteoclasts differentiation and inflammatory bone loss	Tsai-Pei et al. (2011)
Epimedium brevicornu Maxim	Icariin	Osteoclast precursor cell model induced by titanium particle. Osteoclast precursor cells were co-cultured with different concentrations of Icariin for 24, 48, or 72 h	Suppressed the formation of osteoclast. Alleviated osteolysis	Jingfu et al. (2014)
Epimedium brevicornu Maxim	Ikarisoside A (flavonoids)	Macrophage cell model induced by LPS. Macrophage cells were co-cultured with Ikarisoside A for 24 h	Ameliorated the response of inflammatory and oxidative stress in macrophages	Hwa et al. (2008)
Epimedium brevicornu Maxim	Icariine (flvonoid glucoside)	MSCs models of inflammatory and oxidative stress induced by LPS. MSCs co-cultured with different concentrations of Icariin for 3,7, or 21 days	Alleviated bone resorption	Jian et al. (2016)
Salvia miltiorrhiza Bunge	Salvianolic acid B and danshensu	Oxidative stress ovariectomized rat model fed high saturated fat and sucrose. Danshen aqueous extract 600 mg/kg by gavage daily for 12 weeks	Promoted preosteoblast cell proliferation and differentiation. Alleviated bone loss	Dong et al. (2018)
Salvia miltiorrhiza Bunge	Tanshinone IIA and cryptotanshinone	Ovariectomized rat model. Salvia miltiorrhiza extract 1,3,10 or 30 mg/kg by gavage daily for 8 weeks	Prevented bone loss. Inhibited osteoclast number and maturation	Yan et al. (2011), Wenjiu et al. (2022)
Salvia miltiorrhiza Bunge	Tanshinol	Osteogenesis larval zebrafish model induced by dexamethasone. Different concentrations of Tanshinol were fed for 6 days	Promoted differentiation of osteoblast. Increased mineral bone formation	Shiying et al. (2016)
Salvia miltiorrhiza Bunge	Salvianolic acid B	Osteogenesis rat model induced by prednisone. Salvianolic acid B 40 or 80 mg/kg by gavage daily for 12 weeks	Promoted osteoblast differentiation. Alleviated bone loss	Liao et al. (2012)
Pueraria montana var. lobata	Puerarin	Rat model of bone oxidative stress induced by cadmium exposure. Puerarin 200 mg/kg by gavage daily for 5 weeks	Alleviated bone resorption and reconstruction	Xishuai et al. (2022)
Pueraria montana var. lobata	Puerarin	Osteoblast model accompanied by puerarin for 3, 7, and 14 days	Promoted osteoblast proliferation	Shiow-Yunn et al. (2012)
Pueraria montana var. lobata	Isoorientin	Macrophage cell model induced by LPS. Cells were treated with different concentrations of isoorientin (25 nM–100 μM) for 16 h	Alleviated macrophage inflammatory and oxidative stress damage	Kotha et al. (2017)
Gynochthodes officinalis	Morinada officinalis polysaccharide	Tibial dyschondroplasia chicken model induced by tetramethylthiuram disulfide. Morinada officinalis polysaccharide were fed for 21 days	Ameliorated bone injury	Chaodong et al. (2022)
Gynochthodes officinalis	Morinada officinalis polysaccharide	Ovariectomized rat osteoporosis model. Morinada officinalis polysaccharide 100 or 300 mg/kg by gavage daily for 30 days	Increased precipitation of macro elements in bone. Improved bone quality	Zhu et al. (2008)
Gynochthodes officinalis	Morinada officinalis polysaccharide	Ovariectomized rat osteoporosis model. Morinada officinalis polysaccharide 300 mg/kg by gavage daily for 8 weeks	Increased bone mineral density (BMD) and serum level of Cu, Fe, Mg	Kai et al. (2022)
Gynochthodes officinalis	Bajijiasu	Macrophage model co-cultured with RANKL. Macrophage were treated with different concentrations of Bajijiasu for 5 days	Alleviated bone resorption and osteoclast formation	Guoju et al. (2017)
Corethrodendron multijugum	Flavonoids	Bone marrow-derived dendritic cells model co-cultured with LPS. Extraction of dried roots of A. membranaceus were co-cultured with bone marrow-derived dendritic cells for 18 h	Restrained the toxic effect of pro-inflammatory cytokines on bone marrow-derived dendritic cells	Wei et al. (2014)
Corethrodendron multijugum	Astragaloside IV	Iron overloaded mice model induced by intraperitoneal injection of iron dextran. Astragaloside IV at a dose of 40 mg/kg was injected intraperitoneally into the mice in every other day for 4 weeks. Iron overloaded MSC model induced by Ferric ammonium citrate. Cells were treated with different concentrations of Astragaloside IV (25 nM–200 μM) for 2 weeks	Blocked the differentiation of MSCs into adipocytes. Inhibited bone loss. Stimulated the differentiation of MSCs into osteoblasts	Hui et al. (2021)
Corethrodendron multijugum	Astragaloside IV	Oxidative stress macrophage model induced by LPS. Macrophages were co-cultured with Astragali radix extract for 48 h	Suppressed oxidative stress in macrophage	Young et al. (2005)
Corethrodendron multijugum	Isoflavonoids	Oxidative stress macrophage model induced by LPS. Macrophages were co-cultured with Astragali Radix Metabolites for 4 h	Suppressed oxidative stress in macrophage	Li-Jie et al. (2011)
Eclipta prostrata	Total flavonoids of Eclipta prostrata	Ovariectomized rat osteoporosis model. Total flavonoids of Eclipta prostrata 50 mg/kg by gavage daily for 10 weeks	Enhanced antioxidant capacity, Increased bone density, and reduced bone loss	Panyun et al. (2021)
Eclipta prostrata	75% ethanol extraction of Eclipta prostrata (Naringin)	Prednisone-induced oxidative stress osteoporosis mice model. 75% ethanol extraction of Eclipta prostrata 10 g/kg by gavage daily for 12 weeks	Enhanced antioxidant capacity, Increased bone density	Xinyu et al. (2012)
Eclipta prostrata	Curculigoside	Amyloid precursor protein (APP) and presenilin (PS) 1 induced Aβ deposition mice model. Curculigoside 100 mg/kg by gavage daily for 4 weeks	Mitigated bone loss	Lu et al. (2015)
Curculigo orchioides Gaertn	Curculigoside	Iron overloaded mice model induced by iron dextran. Curculigoside (25, 50, or 100) mg/kg by gavage daily for 3 months). Iron overloaded osteoblast model induced by ferric ammonium citrate. Osteoblasts were treated with Curculigoside (10 μM) for 2 h	Prevented bone loss. Alleviated oxidative stress damage to osteoblast	Quanlong et al. (2019)
Curculigo orchioides Gaertn	Curculigoside	Oxidative stress osteoblast model induced by hydrogen peroxide. Osteoblasts were treated with Curculigoside (0.1, 1, or 10 μM) for 2 h	Enhanced the activity of osteoblasts	Ying et al. (2012)
Curculigo orchioides Gaertn	Curculigoside	Macrophage model co-cultured with RANKL. Macrophages were treated with Curculigoside for 72 h	Reduced osteoclastogenesis	Mengqin et al. (2021)
Curculigo orchioides Gaertn	Orcinol glucoside	Aging mice model induced by senescence-accelerated strain. Orcinol glucoside (50 or 100) mg/kg by gavage daily for 10 weeks). Co-cultured macrophage and RANKL model. Macrophages were co-cultured with Orcinol glucoside for 30min, 12, 24, 36, or 48 h	Inhibited osteoclast formation	Wan et al. (2022)
Ligustrum lucidum	Nuezhenide and salidroside)	Premature aging mice model induced by D-galactose (D-gal) and sodium nitrite (NaNO2). Extraction of Ligustrum lucidum 4.9 g/kg was gavage daily for 65 days	Prevented bone loss	Lin et al. (2019b)
Ligustrum lucidum	Nuezhenide and salidroside	Premature aging mice model induced by D-gal. Ligustrum lucidum extract by gavage at 1.75 g/kg/d, 3.5 g/kg//d, or 7 g/kg/d for 20 days. Hydrogen peroxide-induced oxidative stress osteoblast cell model. Osteoblasts were co-cultured with different doses (10–10^-^⁶ mg/mL) of Ligustrum lucidum extract for 24 h	Prevented bone loss. Enhance osteogenesis	Yi et al. (2021)
Ligustrum lucidum	Nuezhenide and salidroside	Ovariectomized rat osteoporosis model. Ligustrum lucidum extract was gavaged at 3.5 g/kg//d for 12 weeks	Prevented bone loss	Lili et al. (2017)
Ligustrum lucidum	Salidroside (SAL)	Ovariectomized rat model. SAL was gavaged daily at 10 mg/kg, 20 mg/kg, or 40 mg/kg for 90 days. Oxidative stress osteoblasts model induced by terttert-butyl hydroperoxide (t-BHP). Osteoblasts were co-cultured with different doses SAL (0.01, 0.1, 1, 10, 100 μM) for 24 or 48 h	Protected osteoblasts from oxidative damage	Yi-Fei et al. (2022)

**TABLE 2:** Pharmacokinetics of active ingredients in Chinese medicine.

Drug	Drug reception route and dose	T1/2	Tmax	Cmax	AUC0→∞	CL	V	References
Icariin	An oral administration to rats at a single dose of 120 mg/kg	0.37 ± 0.17 (h)	1.00 ± 0.31 (ng/mL)	3.51 ± 2.56 (ng⋅ h/mL)	4.14 ± 2.39 (ng⋅h/mL)	43.47 ± 25.10 (L/g)	19.77 ± 16.31 (L/g)	Zhangyang et al. (2022)
Salvianolic acid B	Intravenously administered via tail vein to rats at a dose of 4 mL/kg	4.40 ± 0.55 h	0.03 ± 0.00 h	15304.43 ± 4128.18 ng/mL	2904.36 ± 649.26 (h ng/mL)	0.004 ± 0.002L/(h g)	0.02 ± 0.01 (L/g)	Peng et al. (2019)
Danshensu	Intravenously administered via ear vein to rabbits at a dose of 6.25 mg/kg	31.7 ± 2.5 (min)			1996 ± 356 (ug⋅mL/min)	3.13 ± 0.97 (mL/min/kg)	149 ± 123 (mL/kg)	Zhao et al. (1997)
Tanshinone IIA	Intravenously administered via tail vein to rats at a dose of 4 mg/kg	2.35 ± 0.58 h			109.5 ± 36.5 (ug/h)	36.5 ± 3.2 (h/kg)		Zeng-Jun et al. (2008)
Puerarin	An oral administration to rats at a single dose of 200 mg/kg	0.77 ± 021 h		19.70 ± 4.67 (ug/mL)	56.67 ± 10.65 (mg/h/L)	2.43 ± 1.02 (L/kg/h)	11.40 ± 3.45 (L/kg)	Ran et al. (2011)
Isoorientin	Oral administration to rats at dose of 4 mL/kg	163.4 (min)	25 (min)	35.5 (ng/mL)	3964.1 (ng/min/mL)			Ning et al. (2014)
Astragaloside IV	Intravenously administered via tail vein to rats at a dose of 2 mg/kg	3.01 h			8.89 (ug/h/mL)	0.23 (L/kg/h)	0.64 (L/kg)	Du et al. (2005)
Naringin	An oral administration to humans at a single dose of 135 mg	2.23 h	3.50 h	1937.87 (ng/mL)	9190.68 (ng/h/mL)	241.87 (mL/min)		Kanaze et al. (2007)
curculigoside	Intravenous administration to rats at a dose of 20 mg/kg	121.2 ± 13.4 h	0.17 ± 0.22 h	189,328.5 ± 12,737.6 (ng/mL)	74,323.6 ± 26,484.9 (ng/h/mL)	0.3 ± 0.1 (L/kg/h)	66.1 ± 55.1 (L/kg)	Ting-ting et al. (2015)
salidroside	Intravenous administration to rats at a dose of 50 mg/kg	0.70 ± 0.21 h			7,135.79 ± 1346.40 (h/ng/mL)	1.78 ± 0.36 (L/h)		Na et al. (2012)

### 3.1 Epimedium brevicornu maxim

Epimedium brevicornu Maxim belongs to the Berberidaceae family. A commonly used part for medicine is the dried aerial. It contains abundant nutrients and phytochemicals, such as protein, essential elements, alkaloids, terpenoids, polysaccharides, lignans, and flavonoids (Xiao-Hua et al., 2020). Icariin (ICA) is a flvonoid glucoside active component isolated from Epimedium pubescens. Lipopolysaccharide (LPS) can induce an inflammatory response causing bone resorption and further osteoporosis. A research study elucidated that Icariine restored LPS-induced osteoporosis by suppressing miR-34c level, and further inhibited the pathways of JNK, p38, and NF-kB (Jian et al., 2016). Wear particles can generate chronic inflammation and osteolysis. Therefore, they were thought to be associated with osteoporosis. ICA could treat lesions caused by wear particles by suppressing the expression of inflammatory cytokines TNF-α and IL-1β and the RANK-RANKL signaling pathway (Jingfu et al., 2014). LPS recruits inflammatory cells, produces prostaglandins such as Prostaglandin E2 (PGE2), and synthesizes cytokines IL-6 and TNF-α to mediate inflammatory responses (Shamima et al., 2007). PGE2 and hypoxia-inducible factor-1α (HIF-1α) have the ability to stimulate osteoclast differentiation. ICA suppresses osteoclasts differentiation and inflammatory bone loss by restraining the activation of the MAPK pathway and the expression of PGGE2 and HIF-1α (Tsai-Pei et al., 2011). Inducible nitric oxide synthase (iNOS) is responsible for manufacturing nitric oxide (NO). Excessive NO can cause diversified inflammatory diseases such as inflammatory bone loss (Sunil, 2008; Monia et al., 2009). Ikarisoside A is a natural flavonoid that has antioxidant properties and is in the root of Epimedium brevicornu Maxim Ikarisoside A suppresses the expression of LPS-induced inflammatory mediators NO, TNF-α, and IL-1β as well as iNOS in macrophages via inhibiting p38 kinase and NF-κB signaling (Hwa et al., 2008). Osteoprotegerin (OPG) is a natural antagonist of RANKL. The formation of osteoclast and bone resorption is promoted when the expression of OPG is restrained (Sri and Gallagher, 2014). ICA suppresses the formation and activation of osteoclast which was induced by Ti particles, a wear particle, by decreasing the RANKL/OPG ratio (Hongguo et al., 2015). Excess iron ions are associated with osteoporosis because they facilitate the formation of ROS and are followed by impaired mitochondrial function. Therefore, iron overload has been confirmed as a risk factor for osteoporosis (Viktória, 2017). ICA reverses excess iron ions-induced bone loss by decreasing the production of ROS and ameliorating the impairment of mitochondria (Xingzhi et al., 2019). Iron overload leads to retardation and autophagy of osteoblast progenitors by inhibiting the Phosphatidylinositol 3-kinase (PI3K)/Akt/mammalian target of rapamycin (mTOR) signaling pathway (Wan-Jing et al., 2018). ICA could alleviate mitochondrial dysfunction and ROS production caused by iron overload in MSCs via initiating PI3K/AKT/mTOR and inhibiting MAPK pathways (Xudong et al., 2019), which cause MSCs to differentiate into osteoblasts unceasingly.

### 3.2 Salvia miltiorrhiza bunge

Part of the roots of Salvia miltiorrhiza Bunge [Lamiaceae] has been used in the treatment of cardiovascular disease as a medicine (Chun-Yan et al., 2015). Recently, various studies uncovered that it could be applied to treat inflammatory bone disease, especially osteoporosis. Some researchers have demonstrated that a high-fat-sucrose (HFS) diet will increase ROS and lipid peroxidation which inevitably causes oxidative stress injury (Yanjun et al., 2015). Salvianolic acid B and danshensu have been identified as major active ingredients in Salvia miltiorrhiza Bunge aqueous extract (SMA). A study proved that SMA could act against lipid peroxidation and ROS level in tissues of HFS-fed ovariectomized rats As a consequence, it ameliorates bone loss and promotes preosteoblast cell proliferation and differentiation (Dong et al., 2018). The plasma malondialdehyde (MDA) level represents the plasma lipid peroxidation level, which indirectly reflects plasma oxidative stress (Draper and Hadley, 1990). Ethanol extract of Salvia miltiorrhiza Bunge, the primary components of which are tanshinone IIA and cryptotanshinone, inhibit osteoclast proliferation and maturation via reducing the production of NO and MDA to ameliorate impairment of oxidative stress (Yan et al., 2011). In addition, cryptotanshinone could significantly reduce bone loss by restraining the expression of osteoclast differentiation regulators including cathepsin K, NFATc1, and c-fos (Wenjiu et al., 2022). Dexamethasone (Dex) can lead to glucocorticoid-induced osteoporosis (GIO) by inducing oxidative stress (Fabien et al., 2009; Maria et al., 2011). Tanshinol {D (+)β-3,4-dihydroxyphenyl lactic acid} is one of the water-soluble components of Salvia miltiorrhiza Bunge. Relevant evidence has uncovered that tanshinol could act against oxidative stress and has an inhibitory effect on osteoblastic differentiation induced by Dex via suppressing excessive ROS generation (Shiying et al., 2016). Prednisone is a glucocorticoid like dexamethasone that can induce osteoporosis. It was reported that salvianolic acid B could increase bone mass and prevent prednisone-induced osteoporosis by depressing adipogenesis and stimulating angiogenesis as well as osteogenesis (Liao et al., 2012). Glutathione (GSH) is an antioxidant.

### 3.3 Pueraria montana var. lobata

Pueraria montana var. lobata, the dried root of Pueraria montana var. lobata from the family of Fabaceae, is a perennial winding botanical drug that grows naturally in Southeast Asia. Modern pharmacology has proved that Pueraria montana var. lobata has anti-depression, anti-oxidation, prevention of osteonecrosis, lowering of blood lipids, and other pharmacological effects (Zhen Z. et al., 2013). Cadmium exposure contributes to oxidative injury by reducing the levels of antioxidant glutathione (GSH), and intercellular ROS in osteoblast-like Saos-2 cells (Spenser et al., 2009). Simultaneously, cadmium exposure ameliorates bone density and destructs the microstructural of trabecular bone (Mayra et al., 2021). Puerarin is an isoflavonoid metabolite isolated from the botanical drug puerariae. Previous research demonstrated that puerarin can alleviate oxidative damage from cadmium exposure by regulating autophagy (Xishuai et al., 2022). BMP families can not only facilitate the differentiation of MSCs into osteoblasts and chondrocytes but also promote the differentiation of osteoblast progenitor cells into osteoblasts (Jay et al., 2002). A research study indicated that puerarin promotes the proliferation of osteoblasts probably by initiating bone morphogenetic protein (BMP) and inhibiting the NO pathway (Shiow-Yunn et al., 2012). Cyclooxygenase-2 (COX-2), a variety of inflammatory mediators, mediates inflammatory disease by regulating the oxygenated metabolites of arachidonic acid. Isoorientin is an active component extracted from the tubers of Pueraria tuberosa. It alleviates macrophage inflammatory and oxidative stress damage adequately via decreasing inflammatory mediators such as iNOS and COX-2, inflammatory factors such as TNF-α and IL-1β, and nuclear translocation of NF-KB (Kotha et al., 2017).

### 3.4 Gynochthodes officinalis

Gynochthodes officinalis is a plant belonging to the genus Morinda of the family Rubiaceae. The dried roots are commonly used in medicine. Modern pharmacology has illustrated that Morinda officinalis possesses anti-depression, antioxidant, pro-fertility, and other pharmacological effects (Meng-Yun et al., 2022). The BMP/Smads signaling pathway is highly associated with bone formation and has been identified to affect the differentiation of osteoblasts (Valerie et al., 2016). Morinada officinalis polysaccharide (MOP) is an active ingredient deriving from Morinada officinalis. MOP, consisting mainly of galactose, galacturonic acid, and arabinose, could inhibit osteoclast differentiation, facilitate osteoblast maturation and proliferation, and elevate the antioxidant capacity to withstand bone injury caused by tetramethylthiuram disulfide through the BMP/Smads pathway (Chaodong et al., 2022). Interleukin-6 (IL-6), an interleukin, is both a pro-inflammatory and an anti-inflammatory cytokine. MOP improves bone quality and postpones osteoporosis development by converting IL-6 into an anti-inflammatory cytokine (Zhu et al., 2008). Peroxisome proliferator-activated receptor γ (PPARγ) and its coactivator-1α (PGC-1α) are connected with osteoporosis caused by oxidative stress (Bo et al., 2018; Jian et al., 2018). There is evidence that MOP could inhibit oxidative stress in ovariectomized rats through the hindrance of the PGC-1α/PPARγ pathway to increase the level of reducing agents superoxide dismutase (SOD) and GSH (Kai et al., 2022). This further elucidates that MOP could reduce oxidative stress in ovariectomized rats. Bajijiasu, a variety of dimeric fructose, is extracted from the root of Gynochthodes officinalis. It has been demonstrated that bajijiasu could mitigate oxidative stress induced by RANKL by inhibiting NF-κB and NFATc1 activation. As a result, it is able to attenuate bone resorption and osteoclast formation (Guoju et al., 2017).

### 3.5 Corethrodendron multijugum

Corethrodendron multijugum is a Fabaceae plant widely used to strengthen the body’s natural defenses. Recently, many studies have shown that Corethrodendron multijugum has anti-inflammatory activity and can regulate the growth balance between osteoclasts and osteoblasts (Young et al., 2005; Li-Jie et al., 2011). The p38 pathway regulates the transcription of pro-inflammatory cytokines. Flavonoids isolated from the roots of Corethrodendron multijugum are capable of inhibiting the production of pro-inflammatory cytokines such as IL-6, IL-12, and TNF-α by suppressing the p38 and MAPK pathways. This restrains the toxic effect of pro-inflammatory cytokines on bone marrow-derived dendritic cells (Wei et al., 2014). As we have described before, iron overload is associated with the development of osteoporosis. Astragaloside IV, a tetracyclic triterpenoid saponin extracted from Corethrodendron multijugum, is suggested to block the differentiation of MSCs into adipocytes and bone loss under the condition of iron overload; it also simultaneously stimulates the differentiation of MSCs into osteoblasts (Hui et al., 2021). Aqueous extracted Corethrodendron multijugum (the main component is astragaloside IV) has been proven to suppress the generation of NO and transcription of iNOS mRNA in LPS co-cultured RAW 264.7 macrophages (Young et al., 2005). In summary, we believe that astragalus can treat osteoporosis through its anti-inflammatory and antioxidant properties.

### 3.6 Eclipta prostrata

The dried Eclipta prostrata [Asteraceae] is a perennial botanical drug that is frequently used medicinally to treat musculoskeletal disorders (Zhicai et al., 2022). There is evidence that the total flavonoids of Eclipta prostrata (TFRD) augment the levels of antioxidant SOD and GSH and reduce the generation of ROS. Therefore, we can conclude that TFRD (including naringin, naringenin, and neoeriocitrin) has an antioxidant property. PI3K/AKT is able to promote the transcription of the osteoblast-related gene RUNX2. This article also demonstrated that TFRD can facilitate osteoblast maturation by inhibiting the phosphorylation of the PI3K/AKT pathway. As a result, TFRD actively enhanced antioxidant capacity, increased bone density, and reduced bone loss (Panyun et al., 2021). In a prednisone-induced oxidative stress osteoporosis model, the production of excess oxygen radicals resulted in decreased protein concentration and increased carbyl content in mice, which was reversed by the 75% alcohol concentration of the Eclipta prostrata extract (the main component is Naringin) (Xinyu et al., 2012). Naringin has also been reported to prevent glucocorticoid-induced inflammatory bone loss by inhibiting the levels of the inflammatory factor TNF-α (Chengli et al., 2018). This suggests that the 75% ethanol extraction of Eclipta prostrata has the ability to inhibit oxidative stress induced by glucocorticoids.

### 3.7 Curculigo orchioides gaertn

Curculigo orchioides Gaertn is a tropical and subtropical Hypoxidaceae plant. Dried Curculigo orchioides Gaertn was used in TCM to treat knee arthritis, diarrhea, and leg fatigue (Zhicai et al., 2022). According to a recent study, Curculigo orchioides Gaertn seems to have antioxidant effects as a treatment for osteoporosis. Curculigoside (CCG) is the dominant bioactive metabolite in Curculigo orchioides Gaertn. CCG can not only improve the differentiation and proliferation of osteoblasts but also inhibit osteoclastogenesis by reducing dexamethasone-induced production of ROS and pro-inflammatory cytokines such as interleukin-1β (IL-1β) and TNF-α (Fang-Bing et al., 2015). Amyloid beta peptide (Aβ) deposition causes oxidative damage, contributing to osteoporosis (Carolin et al., 2014). CCG mitigated bone loss following Aβ deposition-induced oxidative damage by reinforcing SOD activity and reducing the generation of pro-inflammatory cytokines IL-6 and TNF-α (Lu et al., 2015). Forkhead Box Protein O1 (FoxO1) enhanced the activity of the antioxidant enzyme SOD after transfer from the cytoplasm to the nucleus. However, the phosphorylation of serine/threonine kinase (AKT) and the overactivation of insulin-like growth factor receptor (IGFR) results in the degradation of FoxO1. CCG counteracts iron overload-induced bone loss principally by inhibiting the IGFR/Akt pathway to increase FoxO1 expression in the nucleus (Quanlong et al., 2019). Orcinol glucoside (OG) is a phenolic glycoside isolated from Curculigo orchioides Gaertn. Nrf2 is associated with the regulation of oxidative stress at the cellular and tissue levels by negatively regulating ROS (Hiroyuki et al., 2016). Keap1 negatively regulates Nrf2 expression by inhibiting the nuclear translocation of Nrf2, which leads to decreased production of antioxidants (including HO-1, GCS, NQO1, and G6PD) associated with Nrf2. Activation of autophagy is connected with osteoclastogenesis. However, this autophagy process can be negatively regulated by the redox-sensitive factor mTOR to restrain osteoclast generation (Xishuai et al., 2019). It has been reported that OG can suppress osteoclast formation, eliminate ROS, up-regulate antioxidant enzyme levels, and inhibit autophagy by activating the Nrf2/Keap1 pathway and mTOR pathway (Wan et al., 2022). In addition, there is a study that demonstrated that CCG can reduce ROS production and osteoclastogenesis by activating the Nrf2 pathway and restraining the NF-KB pathway (Mengqin et al., 2021). Moreover, CCG can reverse the inhibition of hydrogen peroxide in the activity of osteoblasts, the number of mineralized nodules, and the promotion of reactive oxygen generation so as to enhance the activity of osteoblasts and ameliorate oxidative damage by the MAPK and NF-kB pathways (Ying et al., 2012). In conclusion, with a variety of anti-inflammatory and antioxidant active ingredients, Curculigo orchioides Gaertn can be applied to prevent and treat osteoporosis.

### 3.8 Ligustrum lucidum

Ligustrum lucidum (FLL) is a plant of the Oleaceae family, the medical part of which is its ripe, dry fruit, which has been applied to treat diseases associated with aging. In the case of excessive ROS produced by oxidative stress, the deficiency of sirt6 expression will promote and inhibit the formation of osteoclasts and osteoblasts respectively by regulating the NF-kB signaling pathway, thus resulting in osteoporosis (Lin L. et al., 2019). Ligustrum lucidum aqueous extraction (including nuezhenide and salidroside) reverses the adverse effects of oxidative stress. Furthermore, nuezhenide and salidroside inhibit bone loss caused by oxidative damage by enhancing the activity of the antioxidant enzyme superoxide dismutase (SOD) (Yi et al., 2021). NADPH oxidase 4 (Nox4) is an enzyme that controls the oxidative stress response. Upregulated Nox4 may augment oxidative stress and lead to mitochondrial dysfunction (Junya et al., 2010; Carolin et al., 2014). There is evidence that FLL aqueous extraction improves bone mass in ovariectomized mice through the regulation of the Nox4/ROS/NF-kB pathway (Lili et al., 2017). Specifically, FLL aqueous extraction suppresses the expression of Nox4, thus obliterating excessive ROS, and relieving the mitochondrial dysfunction caused by excessive ROS. Bone mass is then increased by regulating the NF-kB pathway. Salidroside (SAL) is an active component derived from FLL. Terttert-butyl hydroperoxide (t-BHP) can induce oxidative stress in osteoblast cells by causing galactose and fatty acid metabolism disorders (Yi-Fei et al., 2022). When galactose metabolism is impaired, it is converted to galactosyl by aldose reductase (AR). Excessive galactosyl in cells leads to the accumulation of free radicals (Dandan et al., 2020), and superfluous AR contributes to the formation of advanced glycosylation end products (AGEs) (Yan-Ying et al., 2013). Subsequently, the accumulation of AGEs increases ROS production, causing mitochondrial dysfunction and cellular inflammation (Ramin and Sorayya, 2018). Furthermore, the disruption of fatty acid metabolism caused by t-BHP can produce oxygen free radicals and lipid peroxides, thus causing oxidative stress damage to osteoblasts. Interestingly, SAL can enhance antioxidant enzyme activity and reduce oxidative stress damage caused by galactose and fatty acid metabolism disturbance by activating Nrf2 (Yi-Fei et al., 2022). As a result, osteoblast cells were protected from oxidative damage caused by t-BHP.

**FIGURE 2:**
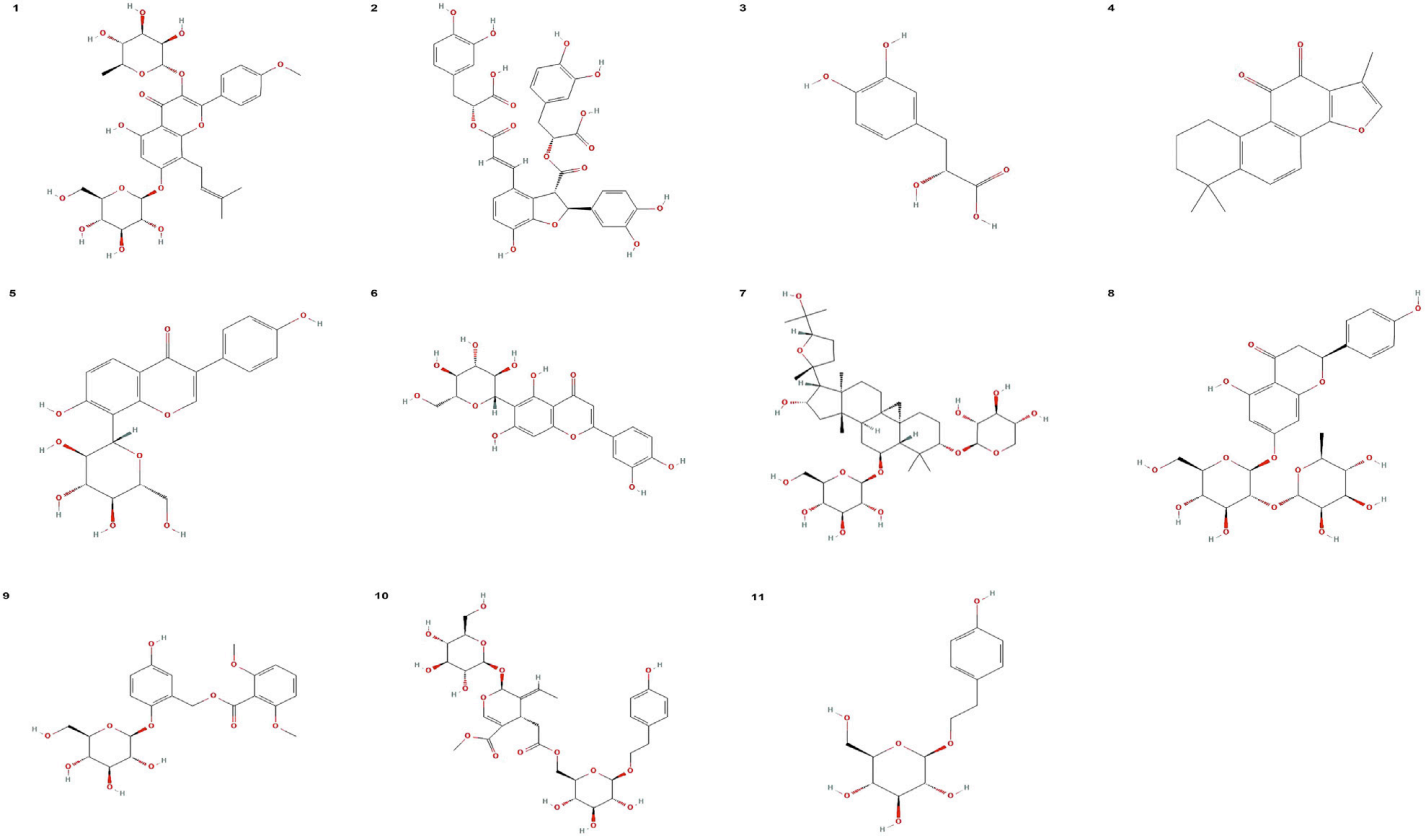
Chemical structure formula of effecttive ingredients of Chinese medicine. (1) Chemical structure formula of icariin. (2) Chemical structure formula of salvianolic acid B. (3) Chemical structure formula of danshensu. (4) Chemical structure formula of Tanshinone IIA. (5) Chemical structure formula of puerarin. (6) Chemical structure formula of isoorientin. (7) Chemical structure formula of astragaloside IV. (8) Chemical structure formula of Naringin. (9) Chemical structure formula of curculigoside. (10) Chemical structure formula of nuezhenide. (11) Chemical structure formula of salidroside. All chemical structure formulas were downloaded from PubChem (https://pubchem.ncbi.nlm.nih.gov/).

In addition to the botanical drugs mentioned above that have therapeutic and preventive effects on inflammation and oxidative stress-induced osteoporosis, there are also many botanical drugs that have not been mentioned. For example, the Chinese botanical drug medicine Psoralea corylifolia can effectively inhibit lipopolysaccharide-induced excess NO production by macrophages and also reduce bone loss by decreasing ROS production (Hisashi et al., 2009; Yinglong et al., 2023). In addition, the water-soluble extract of the Chinese botanical drug medicine Eucommiae Cortex has also been reported to downregulate lipopolysaccharide-induced iNOS, COX-2, and TNF-α in a dose-dependent manner, thus potentially becoming a safe drug for the treatment of inflammatory osteoporosis (Wonil et al., 2017). The chemical structure formula of the active ingredients of Chinese medicine is shown in Figure 2.

## 4 Chinese herbal compound preparation with anti-inflammatory and antioxidant effects in osteoporosis

Although the composition of TCM compound preparations is complicated and the pharmacological active ingredients are difficult to elucidate, they are still widely used as therapeutic drugs due to the advantages of long-term use and few side effects. This review aims to discuss and summarize the anti-inflammatory and antioxidant properties of TCM preparations for the treatment of osteoporosis (Table 3).

**TABLE 3:** Chinese herbal compound in treating osteoporosis.

Name of Chinese medicine compound preparation	Drug composition of compound preparation	Modeling and treatment modalities	Effects on bones	References
Duhuo JiSheng decoction	Achyranthes bidentata Blume, Cinnamomum verum, Asarum heterotropoides F.Schmidt, Gentiana macrophylla Pall, Taxillus chinensis Danser, Angelica biserrata, Eucommia ulmoides Oliv, Smilax glabra Roxb, Saposhnikovia divaricata Schischk, Conioselinum anthriscoides, Panax ginseng C.A.Mey, Glycyrrhiza glabra L, Angelica sinensis Diels, Paeonia lactiflora Pall, Rehmannia glutinosa	Rats ovariectomy model for osteoporosis. Duhuo JiSheng decoction was gavaged daily at 256.5 mg/kg for 3 weeks	Prevented bone loss	Xudong et al. (2022)
Er-xian decoction	Phellodendron chinense C.K.Schneid, Epimedium brevicornu Maxim, Angelica sinensis Diels, Anemarrhena asphodeloides Bunge, Curculigo orchioides Gaertn, Gynochthodes officinalis	*In vitro* model of oxidative stress created by co-culture of MT3T3-E and hydrogen peroxide for 24 h. Osteoblastic cell line MT3T3-E was co-cultured with 15 g/L Er-xian decoction for 24 h	Increased bone density	Nani et al. (2019)
Liuwei Dihuang pills	Dioscorea oppositifolia L, Cornus officinalis, Rehmannia glutinosa, Smilax glabra Roxb, Alisma plantago-aquatica subsp. orientale and Paeonia × suffruticosa Andrews	Network Pharmacology Analysis	Treated postmenopausal osteoporosis	Qingchan et al. (2022)
Qing E pills	Eucommia ulmoides Oliv, Cullen corylifolium, Juglans regia L, and Allium sativum L	*In vivo* osteoporosis model of ovariectomy in rats. Qing E pills were gavaged daily at 4.5 g/kg for 12 weeks	Inhibited activation of osteoclasts. Increased bone mineral density	Hui et al. (2022)
Siwu decoction	Angelica sinensis, Rehmannia glutinosa, Conioselinum anthriscoides ‘Chuanxiong’ and Paeonia lactiflora Pall	*In vitro* model, MT3T3-E was co-cultured with 15 g/L Er-xian decoction for 12 days	Promoted osteoblast maturation and differentiation	Chi-Ming et al. (2013)
Zuo GUI Wan	Cornus Officinalis Sieb, Achyranthis Bidentatae Radix, Lycii Fructus, Cuscutae Semen, deer horn gum, Turtle horn gum, Rhizoma Dioscoreae, Rehmanniae Radix Praeparata	An *in vivo* model of dexamethasone-induced oxidative stress osteoporosis in rats. Zuo GUI Wan was gavaged daily at 1.62 g/kg for 3 months	Delayed senescence of MSCs. Attenuate bone deteriorations	Gengyang et al. (2022), Xiangping et al. (2022)
Yishen Bugu Ye	Cornus officinalis, Achyranthes bidentata Blume, Lycium barbarum L, Cuscuta australis, deer horn gum, Turtle horn gum, Dioscorea oppositifolia L, Rehmannia glutinosa	intraperitoneally injected with 30 mg/kg dexamethasone every other day for 7 weeks to trigger osteoporosis. 5 mL/kg or 10 mL/kg of Yishen Bugu Ye was intraperitoneally injected every other day for 7 weeks	Promoted differentiation of osteoblast. Inhibited differentiation of osteoclast	Yangyang et al. (2020b)
BuShenHuoXue capsule	Epimedium brevicornu Maxim. [Berberidaceae], Eucommia ulmoides Oliv. [Eucommiaceae], Salvia miltiorrhiza Bunge, Conioselinum anthriscoides ‘Chuanxiong’, Paeonia lactiflora Pall, Smilax glabra Roxb, Achyranthes bidentata Blume, deer horn gum, Glycyrrhiza glabra L, Cyperus rotundus L [Cyperaceae]	*In vitro* model, MSCs were co-cultured with BuShenHuoXecapsule for 3 weeks	Protected bone structure. Reduction of MSCs apoptosis	Jia-Cheng et al. (2020)
Yunnan Baiyao	Panax notoginseng [Araliaceae], Cinnamomum camphora [Lauraceae], Cucumis melo L [Cucurbitaceae], Dioscorea oppositifolia L	(0, 1, 10, 100, and 1,000 pg/mL) LPS was used to establish an *in vitro* model of osteoclast inflammation and oxidative stress. Yunnan Baiyao (0, 10, 50, and 200 μg/mL), was co-cultured with osteoclasts for 12, 24, and 48 h	Suppressed osteoclast formation and activation	Xiaobin et al. (2020)
Zhuanggu Zhitong Capsule	Epimedium brevicornu Maxim, Epimedium sagittatum Maxim [Berberidaceae], Lycium barbarum L	*In vivo* osteoporosis model of ovariectomy in rats. Zhuanggu Zhitong Capsule 0.56 g/kg/d was intragastrically administrated, and estradiol was used as a negative control	Recovered bone density	Zhitao et al. (2022)
gushudan	Cnidium monnieri [Apiaceae], Drynaria roosii Nakaike [Polypodiaceae], Salvia miltiorrhiza Bunge, Epimedium brevicornu Maxim	An *in vivo* model of prednisolone 15 mg/kg induced oxidative stress osteoporosis in rats. Gushudan was intragastrically administrated with 30 g/kg/d for 12 weeks	Protected bone from oxidative stress	Shuo et al. (2020)
Fufang Lurong Jiangu capsule	Reynoutria multiflora [Polygonaceae], Cervi Cornu Pantotrichum, Eucommia ulmoides, Placenta Hominis, Angelica sinensis, Panax notoginseng [Araliaceae], Wurfbainia villosa [Zingiberaceae], Testudinis Carapax et Plastrum, Hirudo, Amomi Fructus	100 nM dexamethasone was used to establish an *in vitro* model of MSC inflammation, oxidative stress for 18 days. Fufang Lurong Jiangu was inserted into the cells which had been treated with dexamethasone for 48 h	Improved survival of osteoblast	Wenqi et al. (2020)
Jianpi Qingchang Bushen decoction	Corethrodendron multijugum [Fabaceae], Codonopsis pilosula [Campanulaceae], Alpinia oxyphylla Miq [Zingiberaceae], Portulaca oleracea L [Portulacaceae], Sanguisorba officinalis L [Rosaceae], Dolomiaea costus [Asteraceae], Glycyrrhiza glabra L	An *in vivo* model of piroxicam-induced osteoporosis in mice. Jianpi Qingchang Bushen decoction was intragastrically administered with 16.5 g/kg/d for 14 days	Inhibited bone loss	Ya-Li et al. (2022)
Yishen Zhuanggu Decoction	Cornus Officinalis, Degelatinatum, Cuscuta chinensis Lam [Convolvulaceae], Cyathula officinalis K.C.Kuan [Amaranthaceae], Angelica sinensis, Conioselinum anthriscoides ‘Chuanxiong’, Eucommia ulmoides Oliv, Drynaria roosii Nakaike [Polypodiaceae], Corethrodendron multijugum, Spatholobus Suberectus Dunn, Cyperus rotundus L, Perilla Frutescens	*In vivo* osteoporosis model of ovariectomy in rats. Yishen Zhuanggu Decoction (1 mL/100 g body weight) were intragastrically administered for 12 weeks. As a negative control, an intragastrically administered, 12-week course of calcitrate D600 tablet (5 mg/100 g) was used	Improved bone density	Bole et al. (2022)

As previously mentioned, TNF-α, as an inflammatory factor, can inhibit the differentiation of MSCs into osteoblasts and affect the development and maturation of osteoblasts by inhibiting osteogenic factors such as Runx2. Duhuo JiSheng decoction was first recorded in “Qianjin Yaofang” of Simiao Sun, consisting of 15 traditional Chinese medicines such as Achyranthes bidentata Blume [Amaranthaceae], Cinnamomum verum [Lauraceae], Asarum heterotropoides F. Schmidt [Aristolochiaceae], Gentiana macrophylla Pall [Gentianaceae], Taxillus chinensis Danser [Loranthaceae], Angelica biserrata [Apiaceae], Eucommia ulmoides Oliv [Eucommiaceae], Smilax glabra Roxb [Smilacaceae], Saposhnikovia divaricata Schischk [Apiaceae], Conioselinum anthriscoides [Apiaceae], Panax ginseng C.A.Mey [Araliaceae], Glycyrrhiza glabra L. [Fabaceae], Angelica sinensis Diels [Apiaceae], Paeonia lactiflora Pall [Paeoniaceae], and Rehmannia glutinosa [Orobanchaceae] (Jinyu et al., 2020). It was reported that Duhuo JiSheng decoction could significantly decrease inflammatory cytokine TNF-α in ovariectomized rats to prevent bone loss (Xudong et al., 2022). Er-xian decoction, which includes Phellodendron chinense C.K.Schneid [Rutaceae], Epimedium brevicornu Maxim, Angelica sinensis Diels [Apiaceae], Anemarrhena asphodeloides Bunge [Asparagaceae], Curculigo orchioides Gaertn [Hypoxidaceae], Gynochthodes officinalis [Rubiaceae], is often used for menopause symptomsand is now being applied more and more widely in the treatment of osteoporosis. It has been demonstrated that Er-xian decoction could inhibit the oxidative stress induced by hydrogen peroxide by increasing the content of antioxidant enzymes SOD and GSH, as well as decreasing the levels of nitric oxide and MDA, thereby increasing bone density (Nani et al., 2019). Moreover, Er-xian decoction seems to reduce oxidative stress damage of bone tissue by regulating fatty acid metabolism (Yujie et al., 2023). FoxO1 was discovered to prompt osteoblast proliferation due to its ability to resist oxidative stress (Marie-Therese et al., 2010). Liuwei Dihuang pills were initially recorded in “Children’s Medicine Prescription Strightt Formula” of Yi Qian, consisting of six traditional Chinese medicines such as Dioscorea oppositifolia L [Dioscoreaceae], Cornus officinalis [Cornaceae], Rehmannia glutinosa [Orobanchaceae], Smilax glabra Roxb [Smilacaceae], Alisma plantago-aquatica subsp. orientale [Alismataceae], and Paeonia × suffruticosa Andrews [Paeoniaceae]. Based on the network pharmacological analysis of the target pathway of Liuwei Dihuang pills, it may inhibit the inflammatory response and oxidative stress by acting on the pro-inflammatory cytokines IL-1, IL-6, interleukin-17 (IL-17), and the FoxO1 signaling pathway, thereby treating postmenopausal osteoporosis (Qingchan et al., 2022). Qing E pills, which are composed of Eucommia ulmoides Oliv [Eucommiaceae], Cullen corylifolium [Fabaceae], Juglans regia L [Juglandaceae], and Allium sativum L [Amaryllidaceae], originated from the Song Dynasty book of “Tai Ping Hui Min He Ji Ju Fang” (Nai-Dan et al., 2016). The colonization of the intestinal tract by Firmicutes is related to the intestinal inflammatory response, which mediates the specific expression of pro-inflammatory cytokines TNF-α and Th17. Ultimately, this inflammation will lead to the activation of osteoclasts and induce osteoporosis (Thea and Emilio, 2013; Kyoung-Woon et al., 2015). Qing E pills decrease the level of Firmicutes in the intestinal tract and reverse adverse effects on bone mass (Hui et al., 2022). Siwu decoction was first written about by Daoren Lin during the Tang dynasty in the book “Xianshou Lishang Xuduan Mifang”. Siwu decoction, which is comprised of Angelica sinensis [Apiaceae], Rehmannia glutinosa [Orobanchaceae], Conioselinum anthriscoides ‘Chuanxiong’ [Apiaceae], and Paeonia lactiflora Pall [Paeoniaceae], has been used to treat chronic inflammatory diseases. We can infer that it inhibits osteoporosis due to its anti-inflammatory properties (Chi-Ming et al., 2013). Zuo Gui Wan was first recorded in the “Jingyue Quanshu”, consisting of Cornus officinalis [Cornaceae], Achyranthes bidentata Blume [Amaranthaceae], Lycium barbarum L [Solanaceae], Cuscuta australis [Convolvulaceae], deer horn gum, Turtle horn gum, Dioscorea oppositifolia L [Dioscoreaceae], and Rehmannia glutinosa [Orobanchaceae]. Aging MSCs secrete inflammatory cytokines and proteases that deprive their potential to differentiate into osteoblasts. Fortunately, Zuo Gui Wan can delay the senescence of MSCs to prevent osteoporosis by inhibiting the Wnt/β-catenin signaling pathway (Xiangping et al., 2022). In addition, Zuo Gui Wan alleviates Dex-Induced spinal osteoporosis (Gengyang et al., 2022). It is clear that these TCM preparations play an anti-osteoporosis role through their anti-inflammatory and antioxidant effects.

In recent years, with the continuous development of pharmacology, in addition to the compatibility of traditional fixed TCM, newly developed TCM-compatible preparations have been to applied in the treatment of osteoporosis. New Chinese medicine formulations including BuShenHuoXue capsules, Jianpi Qingchang Bushen decoction, Yishen Bugu Ye, Yunnan Baiyao, and Zhuanggu Zhitong capsules reduce inflammatory bone loss by directly regulating the level of pro-inflammatory factors or indirectly influencing the level of autophagy, thereby regulating the expression of inflammatory factors (Yangyang L. et al., 2020; Jia-Cheng et al., 2020; Xiaobin et al., 2020; Ya-Li et al., 2022; Zhitao et al., 2022). Moreover, Gushudan, Fufang Lurong Jiangu capsules, and Yishen Zhuanggu decoction rely on their antioxidant effect to relieve cells suffering from oxidative stress and thus prevent the occurrence of osteoporosis (Shuo et al., 2020; Wenqi et al., 2020; Bole et al., 2022).

## 5 Pathway analysis of pharmacological effects of network pharmacological on traditional Chinese Medicine

There are numerous active ingredients in TCM. Reasonable screening of active ingredients that can play a therapeutic role and actively search for targeted pathways of drug therapy are conducive to uncovering the mystery of TCM and accurately understanding the whole pharmacological process of TCM. Network pharmacology, which combines computer technology and systems biology, can identify drug targets and active ingredients, and then find disease-related targets and pathways through biological information analysis. Finally, the main pathway of drug treatment of disease will be explored after the intersection of drug targets and disease targets through bioinformatics analysis.

KEGG enrichment analysis (http://www.kegg.jp/) is an integrated database resource for the biological interpretation of genome sequences and other high-throughput data (Minoru et al., 2016). KEGG enrichment analysis showed that an extract of Salvia miltiorrhiza Bunge could significantly regulate the TNF pathway, then inhibit the levels of IL-6, IL-12, and TNF-α, and reduce the inflammatory damage of bone marrow macrophages (Tingting et al., 2021). In addition, the NF-kB pathway has also been verified to be involved in the progression of danshensu against macrophage inflammatory damage (Tingting et al., 2020). Some studies have demonstrated that Morindae Officinalis Radix plays an anti-osteoporosis role by regulating the MAPK pathway to affect the inflammatory response and nitric oxide biosynthesis through a combination of network pharmacology and bioinformation analysis (Shao-Qi et al., 2020). Ecliptae herba may influence the expression of PPARγ by acting on the cancer pathway and the P13K-Akt signaling pathway, thereby associated with protein targets that influence inflammatory responses. The result was the inhibition of glucocorticoid-induced osteoporosis (Fangqing et al., 2022). According to the results of KEGG and gene ontology enrichment analysis, Fructus ligustri lucidi may affect the biological process of the inflammatory response by regulating the P13K-Akt pathway, thus stimulating the osteogenic differentiation of MSCs (Yuanhang et al., 2022). Although there is no direct evidence that Siwu decoction plays an anti-osteoporosis role through a specific pathway, network pharmacological analysis suggests that it can regulate the metabolism of arachidonic acid and thus participate in the inflammatory process. Therefore, it can be inferred that Siwu decoction has certain anti-inflammatory properties (Hua-Li et al., 2021). Moreover, Bushenhuoxue capsules might be connected with reducing the inflammatory response and delaying the biological process of osteoporosis by controlling osteoclast differentiation, phagosome, leukocyte transendothelial migration, and other pathways (Jia-Cheng et al., 2020). Biological analysis indicated that Zhuanggu Zhitong capsules could participate in the metabolic process of ROS and the response to steroid hormones, as well as alleviate bone loss through Akt and apoptosis signaling pathways (Jiangtao et al., 2021). This indicates that Zhuanggu Zhitong capsules could be used to treat oxidative stress-induced osteoporosis depending on the antioxidant activity. 

Although there are many studies that have confirmed the targeting pathways and biological processes as well as the active ingredients of these TCMs with anti-inflammatory and antioxidant activities through network pharmacology, there are still many TCMs, especially TCM compound preparations, which have not been fully explored in network pharmacology and basic research. This makes it difficult to elucidate the mechanism of action of TCM and makes it difficult to standardize the doses and ratios of drugs used; therefore, a clear investigation of the mechanism through network pharmacology and basic experiments is now a top priority.

## 6 Discussion

TCM is a vast resource waiting to be exploited. TCM and its formulations contain abundant anti-inflammatory and antioxidant active ingredients, including flvonoid glucoside, flavonoids, isoflavonoids, saponin, and iridoid glycoside. Icariin from Epimedii herba regulates bone metabolism by suppressing the release of inflammatory cytokines and ameliorating the accumulation of oxidative products. Morinada officinalis polysaccharide from Morindae officinalis Radix has been reported to increase bone mineral density and alleviate bone absorption, attributed to its anti-inflammation and anti-oxidation properties. Curculigoside from Curculiginis rhizome is an antioxidant that alleviates oxidative stress damage in the bone. Although there are a few reports on this subject, the scientific quality of these articles varies as some of them describe the drug preparation methods and pharmacological monitoring methods in detail but some of the reports directly purchased from ready-to-use herbal medicines from reagent dealers and there is no clear mention of the drug composition dose, extraction methods, and active ingredient detection methods This deserves attention in future ethnomedical studies with a view to obtaining more qualitative scientific reports. In addition, a review of the research process of the literature covered in this article showed that a few articles did not explore the drug dose concentrations, only one drug concentration was involved, and negative controls were also lacking. These active ingredients play a role through the multiple targets and multiple pathways. Moreover, with a low price and few adverse reactions, TCM can not only restore the bone microstructure and increase the physiological quality of the bone but also alleviate secondary symptoms such as back pain, and waist and leg weakness (Zhu et al., 2012). However, since the use of TCM and formulations was employed under the guidance of TCM theory, there has not been a unified mode of action for general reference in pharmacology so far, which suggests that more research is urgently needed to fully understand the natural mechanism of drug action and optimize the matching of the drugs.

In conclusion, TCM contains many anti-inflammatory and antioxidant active substances, and its value in the treatment of osteoporosis is beyond doubt. However, since the action mechanism of TCM is multi-targeted and multi-pathway, more pharmacological knowledge is needed to confirm the effectiveness and accuracy of the mechanism of TCM. In brief, it is urgent to develop more effective and safer forms of TCM with clear mechanisms of action. This review elucidates the active ingredients and pathways of action of various herbal medicines, which are relatively wide-ranging but have certain limitations. This review is based on the summary of basic experiments and network pharmacology and lacks a collection of clinical information and clinical verification of the usefulness of the drugs. In addition, the mechanism of action of the active ingredient of the drug on osteoporosis was elucidated in detail, the interaction between the active ingredient of the drug and the active ingredient was not clearly taken into account. Another major limitation is that the efficacy and/or safety of many herbal products relies heavily on low-quality studies that may be limited by the inadequacy or inconsistency of the methods used and the risk of bias in the included studies, thus failing to draw definitive efficacy conclusions. Therefore, more high-quality studies are needed in the field of herbal medicine to clearly determine the efficacy and safety of herbal products.

## Figures and Tables

**Table:** 

**AR**	Aldose reductase
**AGEs**	Advanced glycosylation end products
**AIF**	Apoptosis-inducing factors
**APKA-1**	Apoptotic protein kinase activator-1
**Aβ**	Amyloid beta peptide
**BMP**	Bone morphogenetic protein
**CCG**	Curculigoside
**COX-2**	Cyclooxygenase-2
**JNK**	C-hJun N-terminal kinase
**Cytc**	Cytochrome C
**Dex**	Dexamethasone
**ERK**	Extracellular regulated protein kinases
**FoxO1**	Forkhead Box Protein O1
**FLL**	Fructus ligustri lucidi
**G6PD**	Glucose 6-phosphate dehydrogenase
**GCS**	γ-glutamylcysteine synthetase
**GIO**	Glucorcoticoid-induced osteoporosis
**GSH**	Glutathione
**HO-1**	Heme oxygenase-1
**OH**	Hydroxyl radical
**HIF-1α**	Hypoxia inducible factor-1α
**HFS**	High-fat-sucrose
**IKKs**	IκB kinases
**IL-1**	Interleukin-1
**IL-6**	Interleukin-6
**IL-17**	Interleukin-17
**ICA**	Icriin
**iNOS**	Inducible nitric oxide synthase
**IGFR**	Insulin-like growth factor receptor
**LPS**	Lipopolysaccharide
**MOP**	Morinada officinalis polysaccharide
**MAPK**	Mitogen-activated protein kinase
**MDA**	Malondialdehyde
**MMP9**	Matrix metalloprotein
**MSC**	Mesenchymal stem cells
**mTOR**	Mammalian target of rapamycin
**Nox4**	NADPH oxidase 4
**NF-κB**	Nuclear factor kappa-light-chain-enhancer of activated B Cells
**NFATc1**	Nuclear factor of activated T Cells, cytoplasmic, calcineurin-dependent 1
**Nrf2**	Nuclear factor E2-related factor 2
**NQO-1**	NADPH: quinone oxidoreductase 1
**NO**	Nitric oxide
**OG**	Orcinol glucoside
**OPG**	Osteoprotegerin
**PPARγ**	Peroxisome proliferator-activated receptor γ
**PGE2**	Prostaglandin E2
**PGC-1α**	PPARγ coactivator-1α
**PI3K**	Phosphatidylinositol 3-kinase
**ONOO−**	Peroxynitrite
**RUNX2**	Runt-related transcription factor 2
**ROS**	Reactive oxygen species
**SOD**	Superoxide dismutase
**SMA**	Salvia miltiorrhiza Bunge aqueous extract
**AKT**	Serine/threonine kinase
**SOD**	Superoxide dismutase
**SAL**	Salidroside
**SERMs**	Selective estrogen receptor modulators
**TCM**	Traditional Chinese medicine
**t-BHP**	Terttert-butyl hydroperoxide
**TRAF6**	TNF receptor-associated factor 6
**TRAP**	Triiodothyronine receptor auxiliary protein
**TCMSP**	Traditional Chinese Medicine Systems Pharmacology Database and Analysis Platform
**TFRD**	Total flavonoids of Ecliptae Herba
**TNF-α**	Tumor necrosis factor-α
